# Impact of donor pool size on the variability of platelet lysate-derived extracellular vesicles for regenerative medicine

**DOI:** 10.20517/evcna.2024.05

**Published:** 2024-05-29

**Authors:** Andreu Miquel Amengual-Tugores, Carmen Ráez-Meseguer, Maria Antònia Forteza-Genestra, Javier Calvo, Antoni Gayà, Marta Monjo, Joana Maria Ramis

**Affiliations:** ^1^Group of Cell Therapy and Tissue Engineering (TERCIT), Research Institute on Health Sciences (IUNICS), University of the Balearic Islands (UIB), Palma 07122, Spain.; ^2^Health Research Institute of the Balearic Islands (IdISBa), Palma 07122, Spain.; ^3^Departament of Fundamental Biology and Health Sciences, UIB, Palma 07122, Spain.; ^4^Fundació Banc de Sang i Teixits de les Illes Balears (FBSTIB), Palma 07004, Spain.

**Keywords:** Variability, coefficient of variability, wound healing, platelet lysate-derived extracellular vesicles, platelet concentrate (PC), multiple platelet concentrate (MPC)

## Abstract

**Aim:** The objective of the present study was to determine the variability of platelet lysate-derived extracellular vesicles (pEV), in terms of characteristics and functionality through wound healing assays, when isolated either from platelet concentrates (PC, obtained from 5 donors) or from multiple PC (MPC, that is 50 donors).

**Methods:** pEV were isolated under GMP-like conditions in a clean room using Size Exclusion Chromatography (SEC). The differential characteristics between pEV obtained from PC (PC-EV) or MPC (MPC-EV) were evaluated by means of protein concentration, Nanoparticle Tracking Analysis (NTA), Transmission Electron Microscopy (TEM), and flow cytometry using the MACSPlex™ arrays for surface analysis profiling of EV. The functionality of the isolated pEV was determined in cell culture by metabolic activity and LDH activity determination and through a wound healing assay after 24 h treatment.

**Results:** No significant differences were observed in the pEV characteristics evaluated, whether isolated from PC or MPC. As regards functionality, a higher wound closure percentage was obtained in those pEV pools isolated from PC (5 donors). No differences in the coefficient of variation (CV) were found when comparing all the evaluated variables of pEV derived either from PC (5 donors) or from MPC (50 donors).

**Conclusion:** Our findings challenge the necessity of a larger donor pool for pEV isolation, revealing no significant variations in the analyzed variables of MPC-EV and PC-EV. Notably, our results suggest that, unlike platelet concentrates, a high number of donors is not required to reduce the variability of pEV, showing that the pool of only 5 donors can provide a consistent and reliable therapeutic product.

## INTRODUCTION

Extracellular vesicles (EV) are membranous and nanometric structures with heterogeneous molecular cargo secreted by any cell type that intervenes in cell-to-cell communication^[1]^. This relevant role of EV has sparked interest in the study of its clinical and biotechnological applications^[2]^. Among these applications, one of the extensively investigated areas is their therapeutic potential in regenerative medicine. Since their discovery in 1967 as “platelet dust”, platelet-derived extracellular vesicles (pEV) have shown high potential as a therapeutic asset in this field. They have been suggested as major effectors in the activity of platelet concentrates (PCs)^[3,4]^. Therefore, the study of pEV in tissue regeneration has been one of the main objectives of our group. pEV have been shown to have great clinical translatability, with good results in improving osteogenic potential^[5]^, regenerative effect for gingival and skin wound healing applications^[6-9]^ and osteoarthritis^[10]^. Additionally, its combination with biomaterials for different clinical applications has been explored^[11,12]^. The molecular cargo of pEV, such as proteins and miRNAs, have been postulated as effectors of their regenerative potential^[13,14]^. EV isolated from different sources, such as human umbilical cordon Mesenchymal Stem cells (MSC), induced Pluripotent Stem Cells (iPSC), and Human Umbilical Vein Endothelial Cells (HUVEC), have also been explored for this purpose, as reviewed in^[15]^. In comparative *in vitro* and *in vivo* studies, we have shown that pEV have greater regenerative potential and greater clinical translatability than MSC-derived EV^[10]^.

Platelet concentrates (PCs) used for the production of pEV may have an impact on their therapeutic effect^[16]^. Furthermore, these concentrates not only play a vital role in generating pEV but also have distinct clinical and biotechnological applications. For instance, they are employed as a supplement in culture media for various cell types, including MSC, and have clinical applications in conditions such as osteoarthritis and osteogenesis^[17]^.

The batch-to-batch variability of a therapeutic biological product is one of the limiting factors for its application to clinical practice according to regulatory standards. Indeed, previous studies have demonstrated that there is an important correlation between the number of donors, storage time, and the processing of PC products in their quality control^[18]^. For instance, the number of donors and depletion of leucocytes in Platelet-Rich Plasma can determine the reliability of the final product, in addition to having shown that all these factors can affect the functional activity of these PC^[19]^. One study has determined that 16 donors are the optimal number needed to obtain PC batches with reduced variability in growth factor content^[20]^.

However, to the best of our knowledge, the impact of the number of donors used for PC procurement as a source for pEV isolation has not been evaluated so far. This oversight is significant, given that the original platelets from which pEV are isolated for therapeutic use may vary in their functional characteristics based on the diverse donor pool, potentially influencing the therapeutic efficacy of the final product^[16]^. Thus, in this study, we investigated the batch variability on the characteristics and functionality of pEV isolated from PC obtained from pools with different numbers of donors. We explored whether the number of donors influences both the production of these vesicles as a homogeneous therapeutic product and their characteristics. Blood banks typically prepare PC for patient transfusions. Considering that the standard procedure for obtaining PC involves the pooling of 5 buffy coats from 5 different donors, we compared pEV derived from platelet lysates obtained by pooling 50 buffy coats (MPC-EV) with those obtained from 5 buffy coats (PC-EV), reflecting the number of blood donations used to produce one platelet concentrate at our local Blood Bank (Banc de Sang i Teixits de les Illes Balears). Our analysis aims to understand how the varied donor numbers across different batches may affect the characteristics of the obtained pEV intended for quality control in a specific therapeutic product, along with its functionality and potential implications for clinical translation.

## METHODS

### Platelet concentrates procurement

Platelet concentrates MPC and PC were provided by the IdISBa Biobank, with the approval of the Ethics Committee (IB1955/12 BIO ref. 02/2021) after ethical approval of the project by the CEI-IB (IB 4453/21 PI). Buffy coats from 5 blood donations from 5 different donors are used to produce one platelet concentrate (PC) at the Blood Bank. Once pooled, the 5 buffy coats were pooled, washed with 0.9% NaCl (Braun, city, country), and centrifuged at 651 × *g* for 10 min, obtaining PC. Each MPC was obtained by adding equal volumes of 10 PCs. Here, 9 PC and 10 MPC were prepared to perform the comparative experiments. PC and MPC were lysed by applying three freeze/thaw cycles (-80 ºC/37 ºC) to the platelet concentrate, a freeze/thaw cycle (-80 ºC/55 ºC) for pasteurization, followed by centrifugation at 5,050 × *g* for 20 min and filtration through 40 μm porous membrane filters (Sartorius, Goettingen, Germany). Then, all these lysed PC and MPC were centrifuged, firstly at 1,500 × *g* for 15 min at 4 °C and secondly at 10,000 × *g* for 30 min at 4 °C, keeping the supernatant after each centrifugation. Subsequently, the supernatant was filtered successively through 0.8 and 0.2 µm porous membrane filters (Sartorius).

### pEV isolation

pEV from lysed MPC and PC were isolated by Size Exclusion Chromatography (SEC), loading 10 mL of each filtered PC or MPC into the chromatography loop. A commercial column HiPrep 26/60 SephacrylS 400HR (Cytiva, Marlborough, MA, USA) connected to an ÄKTA go system (Cytiva) coupled with a fraction collector F9-R (Cytiva) was set to allow a flow rate of 1.3 mL/min using 0.9 % NaCl (Braun), for MPC7, MPC8, and MPC9, and Plasmalyte pH 7.4 (Viaflo, Italy) for the rest of MPC and PC. Fractions 8, 9, and 10 were pooled and used as pEV preparations [Supplementary Figures 1 and 2]. This process was carried out under GMP-like conditions in the clean room of the Fundació Banc de Sang i Teixits de les Illes Balears.

### Protein quantification

Total protein content was determined using the Pierce™ BCA Protein Assay Kit (Thermo Scientific, Waltham, MA, USA) following the manufacturer’s protocol.

### Determination of lipid content of pEV

Lipid content was determined as previously described^[21]^. Briefly, a volume of 200 µL of 96 % sulfuric acid (Molar Chemicals) was added either to 40 µL of liposome standards or to 40 µL pEV suspended in Plasmalyte pH 7 (Viaflo, Italy). Test tubes were incubated at 90 °C in a fume hood for 20 min. Tubes were cooled down to RT by placing them for at least 5 min in an ice bath, and 120 µL of phospho-vanillin reagent was added to each tube and vortexed. Next, 280 µL of each sample was transferred to a 96-well plate (Thermo) and the color reaction was allowed to develop for 1 h at 37 °C. Absorbance was determined at 540 nm.

### Nanoparticle tracking analysis

The number of particles and their size distribution were analyzed using Nanoparticle Tracking Analysis with Nanosight NS300 (Malvern Instruments, Malvern, UK). Data were analyzed with NTA 3.2 Dev Build 3.2.16 Software.

### Flow cytometry

Isolated pEV from all lysed PC and MPC were processed with the MACSPlex Exosome Kit (Miltenyi Biotec, Germany). The manufacturer’s instructions were followed, loading 17.5 µg of protein for each sample. The fluorescence measurement was performed using BD FACSVerse (BD Biosciences, Franklin Lake, NJ, USA) flow cytometer. For data analysis, the fluorescence in each sample was normalized using the average of the medians of the CD9, CD63, and CD81 fluorescence of each sample in the APC-H channel.

### Cell culture

Immortalized human gingival fibroblasts-hTERT-hNOF (ihGF, Applied Biological Materials Inc, Richmond, Canada), grown at 37 °C and 5 % CO_2_, were used in cell culture experiments. Cells were maintained in DMEM low glucose (Biowest, Riverside, UK) and Ham’s F12 (Biowest) in a 2:1 proportion, supplemented with 10 % (v/v) FBS (Capricorn, Düsseldorf, Germany) and 100 µg/mL penicillin, and 100 µg/mL streptomycin (Biowest).

### Wound healing assay

Cells were seeded at a density of 30,000 cells per well in a 48-well plate in three independent experiments, each conducted with triplicates. After three days, when cells had reached confluence, the cell medium was replaced by a medium containing 1 % EV’s-depleted FBS (ultracentrifuged at 120,000 × *g* for 18 h at 4 °C). After 24 h, the wound was created using a sterile pipette tip, images were taken using a bright-field inverted microscope (Nikon Eclipse TS100, Nikon, Tokyo, Japan), and cells were treated with 1 × 10^10^ particles per well of each MPC and PC-EV. After 24 h, imaging was repeated. For comparison between sources, cells were treated with the equivalent amount of protein corresponding to that number of particles.

Images were analyzed with Image J software (NIH, Bethesda, Maryland, USA) using a wound healing assay plugin^[22]^. Images were filtered at 32 pixels using the variance method and wound area was obtained. The percentage of wound closure was calculated using the following equation:

**Figure:**
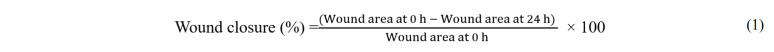


### Metabolic activity assay

To measure metabolic activity 24 h after treatment with pEV, we used the Presto Blue® reagent (LifeTechnologies/Thermo Fisher Scientific, Basel, Switzerland) following the manufacturer’s instructions. Negative control cells (wounded and non-treated) were considered the 100% control of metabolic activity.

### Cytotoxicity

To determine the biocompatibility of pEV, lactate dehydrogenase (LDH) activity released to cell culture media was measured 24 h after treatment with the commercial Cytotoxicity Detection kit (Roche Diagnostics, Manheim, Germany) following the manufacturer’s instructions. For cytotoxicity calculation, cells treated with 0.1 % Triton X-100 were used as a high control (100 % of cell death), and control cells (non-wounded and non-treated) as a low control (0 % of cell death). The percentage of cytotoxicity was calculated using the following formula:

**Figure:**
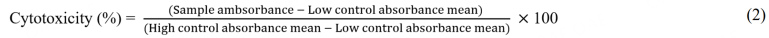


### Statistical analysis

For all the experiments, PC-EV batches (*n* = 10) and MPC-EV (*n* = 9) were evaluated. All data are presented as mean values ± SEM. In addition, for each quantitative variable evaluated, the coefficient of variation of PC and MPC was calculated. The Shapiro-Wilk test was done to assume parametric or non-parametric distributions for the normality tests. Differences between groups were assessed by the Mann-Whitney test or by Student’s *t*-test, depending on their normal distribution. When more than two groups were compared, ANOVA and Student’s *t*-test post-hoc comparison were employed. The results were considered statistically significant at *P* values < 0.05 and compared with *t*-test statistical comparison. These analyses were performed using GraphPad Prism 8 Software (La Jolla, CA, USA).

## RESULTS

### Characterization of the pEV isolated from MPC and PC

**Figure 1:**
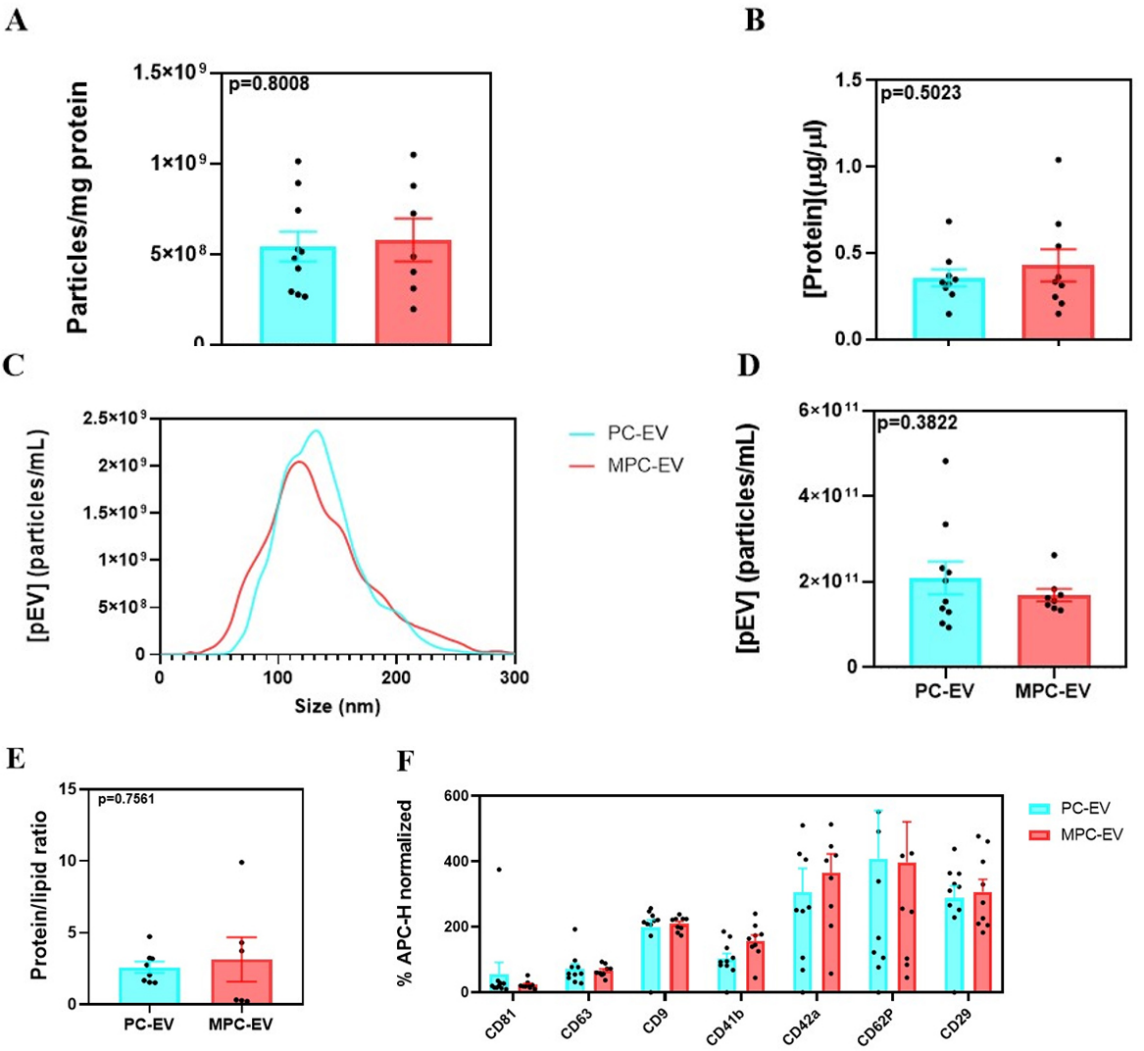
Characterization of the pEV isolated from PC and MPC. (A) Purity of pEV (number of particles per mg of protein) isolated from MPC and PC; (B) Protein concentration of pEV isolated from MPC and PC; (C) Particle size distribution diagram, comparing pEV isolated from PC (blue) and MPC (red); (D) Particle yield of pEV isolated from PC and MPC; (E) Protein/lipid ratio of pEV isolated from MPC and PC; (F) Comparison of mean normalized fluorescence of the AMC-H channel of PC and MPC pEV for different membrane markers; for each sample, three technical replicates were evaluated. Values represent the mean ± SEM. For all the analyses, 10 PCs (*n* = 10; pooling 5 different donors each), 9 MPCs (*n* = 9, pooling 50 different donors each), and 6 MPCs (*n* = 6, in the protein/lipid ratio) were used. Results were statistically compared by *t*-test. For Figure 1A - E, *P*-values are shown in the graph. For Figure 1F, *P* values for membrane markers were CD81 (*P* = 0.966), CD63 (*P* = 0.999), CD9 (*P* = 0.998), CD41b (*P* = 0.383), CD42a (*P* = 0.894), CD62 (*P* > 0.999) and CD29 (*P* = 0.996). PC: platelet concentrate; MPC: multiple platelet concentrate; pEV: platelet lysate-derived extracellular vesicles.

Similar characteristics were observed for pEV isolated from MPC and PC, as shown in Figure 1A and B; purity (particles/mg protein) and protein (µg/µL) were alike in both groups, also showing similar variability, so was particle size distribution, with particle sizes that range from 50 to 250 nm [Figure 1C]. No differences were observed in the particle concentration yield or in the comparison of the protein/lipid ratio for PC compared to MPC [Figure 1D and E].

The analysis of pEV membrane markers through flow cytometry using the MACSPlex Exosome Kit resulted in a higher fluorescence intensity obtained for those membrane markers corresponding to EV and platelet markers (CD29, CD9, CD42a, CD62P, CD41b, CD63, and CD61). No significant differences were observed between PC-derived and MPC-derived pEV for these markers [Figure 1F]. The rest of membrane markers that are evaluated with the MACSPlex Exosome Kit were not detected in our samples (markers of neutrophils-CD45, CD142, CD24-, markers of monocytes -CD14, HLA-Ds-, markers of macrophages-CD40, CD86, CD1c, CD11c, CD209-, markers of T-Cells-Cd2, CD3, CD4, CD8-, natural killer marker-CD56-, activated T cells markers-CD69, CD25-, B cells markers-CD19, CD20-, endothelium markers-CD31, CD146, CD105-, epithelium marker-CD326-, stem cells markers -SSEA-4, CD133_1-, multiple cells markers- HLA-A, CD44, CD49e-, Adipocytes/Parathyroid/Cancer marker-ROR1-, melanocytes/Smooth muscle cells/Cancer marker -MCSP-, controls-REA C, IgG1 C-)^[23]^.

As shown in Supplementary Figure 1, pEV were enriched for CD9 and CD63 tetraspanins in comparison with their PL sources. The contamination of negative marker albumin was similar in both types of pEV in the same way as the HSC70 protein contained in the lumen of EV. In addition, MPC-EV and PC-EV were detected in TEM with uranyl acetate staining.

### Functionality of pEV isolated from MPC and PC

**Figure 2:**
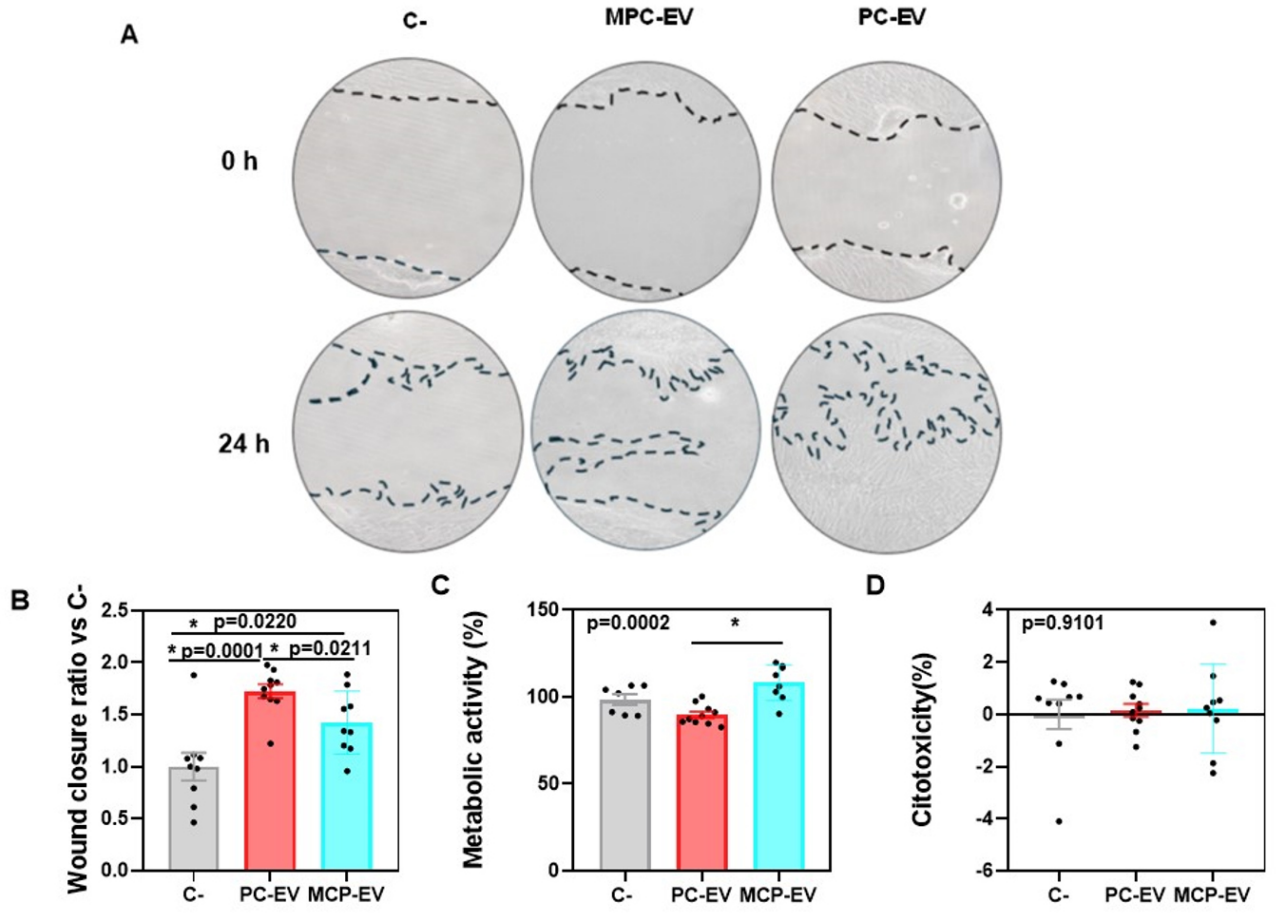
Comparison of the functionality of the pEV obtained from PC and MPC. (A) Representative images of wound healing assay at the moment of treating cells (0 h) and after 24 h; (B) Wound closure ratio after 24 h of wound treatment with pEV obtained from the relationship between wound closure of the treatment with pEV and its negative control using the formula detailed in the material and methods section; (C) Metabolic activity after 24 h of wound treatment with pEV, negative control cells (wounded and non-treated) were considered 100% of the metabolic activity; (D) Cytotoxicity assay of cells treated with pEV isolated from PC and MPC after 24 h of wound treatment with pEV. LDH activity evaluated on the medium of cells that were not wounded was considered 0 % cytotoxicity and LDH activity evaluated on the medium of cells treated with 1% Triton-X was considered 100% cytotoxicity. For all analyses, 10 PCs (*n* = 10; pooling 5 different donors each) and 9 MPCs (*n* = 9, pooling 50 different donors each) were used for *in vitro* treatments. Values represent the mean ± SEM. The differences between groups were determined using ANOVA and Student’s *t*-test post-hoc comparison. PC: platelet concentrate; MPC: multiple platelet concentrate; pEV: platelet lysate-derived extracellular vesicles. *P < 0.05

Wound closure ratios [Figure 2A] of pEV isolated from PC were significantly higher than those isolated from MPC [Figure 2B]. In contrast, higher metabolic activity was observed in cells treated with pEV obtained from MPC compared to those obtained from PC [Figure 2C]. No differences were found in cytotoxicity among both groups, which was similar to that of the low control group (0%) [Figure 2D].

### Variability comparison

**Figure 3:**
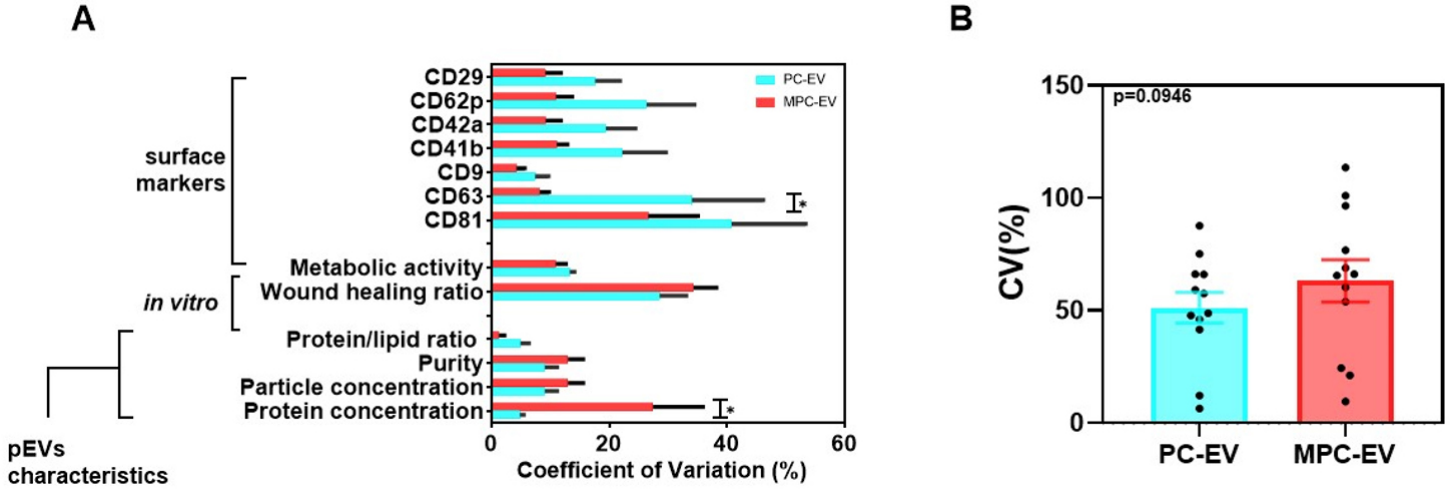
Variability comparison in the evaluated variables of MPC-EV and PC-EV. (A) Coefficient of variation (%) from each variable evaluated for pEV isolated from PC and MPC, including pEV characteristics, *in vitro* functionality results, and surface markers profile. Values represent the mean of CV ± SEM. 10 PCs (*n* = 10; pooling 5 different donors each) and 9 MPCs (*n* = 9, pooling 50 different donors each) were used for variability analyses; (B) Global differences in CV (%) of PC-EV and MPC-EV of all the variables evaluated represented as the mean of the CV ± SEM (*n* = 12). The differences between the parametric means were determined using the *t*-test statistical method. Significant differences are expressed by *P < 0.05. In Figure 3A, *P* values for PC-EV *vs.* MPC-EV were: Protein concentration (*P* = 0.4062), particle concentration (*P* > 0.9999), purity (*P* > 0.9999), protein/lipid ratio (*P* > 0.9999), wound healing ratio (*P* > 0.9999), metabolic activity (*P* > 0.9999), CD81 (*P* = 0.5766), CD63 (*P* = 0.0122), CD9 (*P* > 0.9999); CD41b (*P* = 0.8835); CD42a (*P* = 0.9303), CD62p (*P* = 0.4880 ), and CD29 (*P* = 0.9834). PC: platelet concentrate; MPC: multiple platelet concentrate; pEV: platelet lysate-derived extracellular vesicles.

Examination of variability, represented by the coefficient of variation (CV) [Figure 3], revealed disparities between the variables analyzed in PC-EV and MPC-EV. Specifically, higher variability in CD63 expression was observed in PC-EV, while higher variability in protein content was observed in MPC-EV. On the other hand, no significant differences were evident in the variability analysis for the rest of the variables. When extending our evaluation to the analysis of global variability [Figure 3B], which encompasses the mean variability of all the variables studied, no substantial differences were detected between the conditions analyzed.

## DISCUSSION

Here, we investigated the complex regulatory framework surrounding pEV as a therapeutic blood-derivative product, probing into the impact of donor number on the variability of pEV characteristics. Our findings challenge the necessity of a larger donor pool, revealing no significant variations in the analyzed variables when differences between MPC-EV and PC-EV were evaluated, in contrast to what has been seen when PL are used instead of FBS for stem cell culture^[24]^. Notably, our results suggest that extended waits for donor recruitment can be avoided without compromising the quality and reproducibility of pEV products, proposing a more efficient approach.

PC or MPC manufacturing conditions may have an impact on the therapeutic effect of pEV^[16]^. Furthermore, these concentrates not only play a vital role in generating Pev but also have distinct clinical and biotechnological applications. For example, they are utilized as additives in culture media for diverse cell types, including MSCs, and have clinical relevance in conditions such as osteoarthritis and osteogenesis^[17]^. Taking this into account, one of the most controversial aspects of the use of PC and their derivatives in clinics is the number of donors needed to obtain a homogeneous therapeutic product with low variability through several production batches. As it is known and given in different studies, the number of donors is directly related to the homogeneous content of growth factors for these PC applications^[20]^. Thus, the study of the number of donors necessary to produce pEV, as a product derived from these PCs, should also be evaluated since, to the best of our knowledge, this is the first time it has been reported.

Both pEV groups, isolated either from 10 production batches of PC-EV or from 9 batches of MPC-EV, showed similar purity (particles/mg protein) values to those previously reported for platelet-derived EV^[25]^. The protein/lipid ratio obtained was higher than those obtained for other EV samples^[26]^, but it was in line with those described for pEV^[27]^. However, direct comparison is limited due to differences in the isolation method or even the lipid quantification technique. Moreover, both PC-EV and MPC-EV showed enrichment of tetraspanins CD9 and CD63 compared to the original PL, as well as the presence of luminal EV markers such as HSC70. Additionally, both pEV groups exhibited typical EV characteristics as confirmed by NTA and TEM, following the guidelines of the International Society of Extracellular Vesicles (ISEV)^[1]^. In the same way, albumin and lipoprotein contaminations are equally probable in both cases. All these characteristics show that the quality controls based on this characterization assays would not vary regardless of whether they are isolated from MPC or PC. These results were confirmed by the analysis of membrane markers using the MACSPlex kit. Although there was no notable difference in the expression of membrane markers between MPC-EV and PC-EV, notable differences could be seen between the global expression of these markers. For instance, CD9, one of the fundamental EV tetraspanins, exhibited the highest global expression levels in the evaluated pEV compared to other fundamental tetraspanins. In contrast, CD81 was nearly undetectable, a finding consistent with our previous validations within our research group and also reported by others^[27]^. Furthermore, it was notable that other pEV membrane markers such as CD29, CD42a, CD62P, CD41b, and CD63 are homogeneously present in both MPC-EV and PC-EV. Those are platelet-related membrane markers, and their function is directly associated with their regenerative potential. Indeed, CD42 is a protein that promotes coagulation, platelet adhesion, and angiogenesis^[28]^. CD29 has functions related to hemostasis, while CD62P can intervene in the inflammatory process associated with wound healing^[29]^. Thus, these membrane markers play an important role in the wound healing process, thereby reinforcing the consistency of our *in vitro* results with the observed expression of these markers. It is important to note the lack of expression of other membrane markers included in the MACSPlex kit detection, since pEV showed null expression of those non-specific pEV markers (not shown in Figure 1E). It has previously been described that not all platelet-derived EV subpopulations express CD63^[30]^; this observation could explain the high variability that we have found here, both on the dot blots and on the MACSPlex evaluation, mainly in PC-EV samples. When evaluating this marker, lower variability is observed when pEV are obtained from a pool of a higher number of donors, which would be in agreement with the lower functional variability described on platelet lysates, but we have not observed that on the functional studies performed of pEV.

Although there were minimal differences in the characteristics of pEV isolated from PC and those isolated from MPC, there was a clear difference between their wound closure percentage and metabolic activity. These findings suggest that a high number of donors is not necessary for the application of pEV as a therapeutic agent in regenerative medicine to reduce variability, at least with respect to the migration of human fibroblasts. In fact, a higher wound closure rate was observed for PC-EV. This observation can be attributed to the significantly shorter collection time for starting material (PL) in the case of 5 donors compared to 50. This discrepancy is partly explained by the heightened sensitivity of pEV to storage conditions and storage cycles^[31]^. The conservation of EV in general and their sources is a hotspot in research dealing with these EV. Therefore, we suggest that a smaller number of donors may be sufficient for the reliable effectiveness of pEV, which are the most potent fraction of the platelet secretome. This approach also favors the conservation of these pEV in their natural source. As for the metabolic activity results, although there is a difference between cells treated with PC-EV and MPC-EV, neither group showed a significant variation compared to the control. The fact that PC-EV showed a higher closure rate and lower metabolic activity compared to MPC-EV may be explained by the fact that PC-EV treatment induces higher cell migration while MPC-EV induces higher cell proliferation, both of which contribute to wound healing^[32]^. However, some studies have previously confirmed that migration occurs within the first 24 h of treatment^[33,34]^, so evaluation of both wound closure and metabolic activity should be determined at longer time points to confirm whether these differences would still be found.

In the same way, the variability analyses did not meet expectations. As mentioned earlier, it was expected that a larger number of donors would result in lower variability compared to pEV isolated from a smaller number of donors^[19,35]^ leading to a more homogeneous content and cargo. In previous studies, the optimal number of donors was determined in order to reduce the variability and maintain the concentration of growth factors, while here, we are evaluating the variability on pEV characteristics and functionality^[20]^. However, other variables apart from the number of donors could also affect variability, such as gender, age, or patient’s comorbidities^[35]^.

As a main limitation of this study, when looking for an approach to the application of pEV in regenerative medicine, it is important to note that the results in 2D cultures are of limited extrapolation, and more functional, 3D *in vitro* models or *in vivo* studies would be necessary to confirm the differences observed in the *in vitro* assays described above^[36]^. We would also point out that the optimal number of donors used as a source of pEV should be evaluated for each intended application, as different functional assays may be required, and a different outcome may be obtained. In addition, there is a lack of studies on the long-term effects of pEV-based therapies, which should be addressed in the future. Another limitation of our study is, of course, that we have not evaluated all possible variables and there remains the possibility of various confounding factors that could affect the results obtained.

It Is not beyond our understanding that PCs are commonly used in tissue banks and research institutes as a supplement to MSC and other clinical applications^[37]^. These applications need a higher number of donors to achieve bath-to-batch reliability^[16]^. This is further supported by some unpublished results obtained at the Tissue Bank of the Balearic Islands. Thus, surprisingly, our results contrast with what we expected. It was foreseeable that a higher pEV variability would have been observed when a lower number of donors was used for its isolation. Nevertheless, the studies conducted here do not consider the direct use of platelet concentrates; instead, they focus on the use of pEV, which represent the purest and the most potent fraction of the platelet secretome^[4]^. This raises the possibility that it could also be used as a reliable product of MSC culture media. In summary, our study implies that there is no need to recruit large numbers of donors, minimizing storage time and regulatory concerns. The use of 5-donor platelet pools (PC) for pEV isolation for regenerative applications proves beneficial by maintaining batch-to-batch reliability.

## DECLARATIONS

### Availability of data and materials

We have submitted all experimental relevant data of our experiments to the EV-TRACK knowledgebase (EV-TRACK ID: EV240011) (Van Deun *et al.* EV-TRACK: transparent reporting and centralizing knowledge in extracellular vesicle research. *Nature methods* 2017;14:228-32). We also share relevant data in figshare repository (https://figshare.com/) (https://doi.org/10.6084/m9.figshare.25341640.v1).

### Copyright

© The Author(s) 2024.
