# Supplementary material for: Impact of donor pool size on the variability of platelet lysate-derived extracellular vesicles for regenerative medicine
Source: Extracell Vesicles Circ Nucl Acids. 2024 May 29;5(2):259–70. doi: 10.20517/evcna.2024.05 (PMC11651878; doi:10.20517/evcna.2024.05)
Supplement: Supplementary file 1 [file evcna-5-2-259-SupplementaryMaterials.pdf]

XX *et al. Extracellular Vesicles and Circulating Nucleic Acids* Year;Volume:Number
DOI: 10.20517/2574-1209.xxxx.xx

Extracellular Vesicles and Circulating Nucleic Acids

Supplementary Materials

Impact of donor pool size on the variability of platelet lysate-derived extracellular vesicles for regenerative medicine

Andreu Miquel Amengual-Tugores[1,2,3], Carmen Ráez-Meseguer[1,2,3], Maria Antònia Forteza-Genestra[1,2], Javier Calvo[1,2,4], Antoni Gayà[1,2,4], Marta Monjo[1,2,3], Joana Maria Ramis[1,2,3]

[1]Group of Cell Therapy and Tissue Engineering (TERCIT), Research Institute on Health Sciences (IUNICS), University of the Balearic Islands (UIB), Palma 07122, Spain.
[2]Health Research Institute of the Balearic Islands (IdISBa), Palma 07122, Spain.
[3]Departament of Fundamental Biology and Health Sciences, UIB, Palma 07122, Spain.
[4]Fundació Banc de Sang i Teixits de les Illes Balears (FBSTIB), Palma 07004, Spain.

Correspondence to: Dr. Marta Monjo, Department of Fundamental Biology and Health Science, University of Balearic Islands, Cra. de Valldemossa, km 7,5, Palma 07122, Spain. E-mail: marta.monjo@uib.es; Dr. Joana Maria Ramis, Department of Fundamental Biology and Health Science, University of Balearic Islands, Cra. de Valldemossa, km 7,5, Palma 07122, Spain. E-mail: joana.ramis@uib.es

ORCID: Marta Monjo (0000-0002-2731-9527), Joana Maria Ramis (0000-0001-9109-8362)

Suplementary Material and Methods

TEM

EV samples were mixed 1:1 with 4 % formaldehyde (Sigma-Aldrich, Sant Louis, MO,

© The Author(s) 2020. Open Access This article is licensed under a Creative Commons Attribution 4.0 International License (https://creativecommons.org/licenses/by/4.0/), which permits unrestricted use, sharing, adaptation, distribution and reproduction in any medium or format, for any purpose, even commercially, as long as you give appropriate credit to the original author(s) and the source, provide a link to the Creative Commons license, and indicate if changes were made.

USA) and 10 µL were fixed on copper Formvar-Carbon coated grids (Ted Pella, CA, USA) for 20 min. These grids were washed with PBS and incubated with 1 % glutaraldehyde (Sigma-Aldrich) for 5 min. Finally, grids were washed with deionized water and air dried. To contrast samples, grids were stained with 2 % uranyl acetate (Sigma-Aldrich) for 1 min, washed three times with deionized water, and then air dried. Images of EVs were taken with a transmission electron microscope Talos F200i (Thermo Fisher Scientific, Waltham, MA, USA)

Dot Blots (DB)

To detect typical EV markers (CD9 and CD63), 2.5 µg of protein from each sample were loaded directly onto a nitrocellulose blotting membrane (GE Healthcare Life Sciences, Germany). To detect albumin as a non-EV markers, pEV were lysed with a lysis buffer (NaH 1 M, Na3VO4 100 mM, Na-pirophospate 100 mM, Tritón-X 10%, protease inhibitor 10 X). Then, 5 µg protein were loaded for this protein onto a nitrocellulose blotting membrane (GE Healthcare). Membranes were blocked with TBS Odyssey Intercept Blocking Buffer (LI-COR, Lincoln, NE, USA) and incubated overnight at 4 °C with the following primary antibodies: anti-human CD9 monoclonal antibody (clone Ts9 diluted 1:2,000, Abcam, Cambridge, UK) and anti-human CD63 monoclonal antibody (clone TS63, diluted 1:2,000, Abcam), diluted with TBS Odyssey-0.1 % Tween. Lysed samples were incubated with anti-human albumin monoclonal antibody (clone AL-01, Human Serum Albumin Monoclonal Antibody, diluted 1:1,000, Invitrogen, Carlsbad, CA, USA). Then, all the membranes were incubated for 1 h with IRDye® 800CW Donkey anti-Mouse (diluted 1:8,000, LI-COR) secondary antibody. DiGit Blot scanner, and Image Studio- Digits Software 4.0 (LI-COR) were used for membrane exposure and image processing.

Western Blot

pEV samples were lysed with RIPA × 1 (Millipore, Burlington, MA, USA) and prepared with reducing loading buffer (with β-mercaptoethanol) to detect HSC70 and loaded with the same amount of protein (15 µg) in a 10% SDS-PAGE gels. Proteins

were transferred onto nitrocellulose membrane (GE Healthcare) by humid transference, TBS Odyssey Intercept Blocking Buffer (LI-COR) and incubated overnight with HSC70 primary antibody (diluted 1:1500, Santa Cruz Biotechnology, Dallas, TX, USA). Then, all the membranes were incubated for 1 h with IRDye® 800CW Donkey anti-Mouse (diluted 1:8,000, LI-COR) secondary antibody. DiGit Blot scanner, and Image Studio- Digits Software 4.0 (LI-COR) were used for membrane exposure and image processing.

In order to confirm the correct transferring of the proteins, membranes were then incubated with 0.2% (w/v) Ponceau S (Sigma-Aldrich) in 3% (v/v) acetic acid solution (Sigma-Aldrich) for 5 min. Then, membranes were washed with deionized water, images were taken, and washed finally with TBS for 5 min.

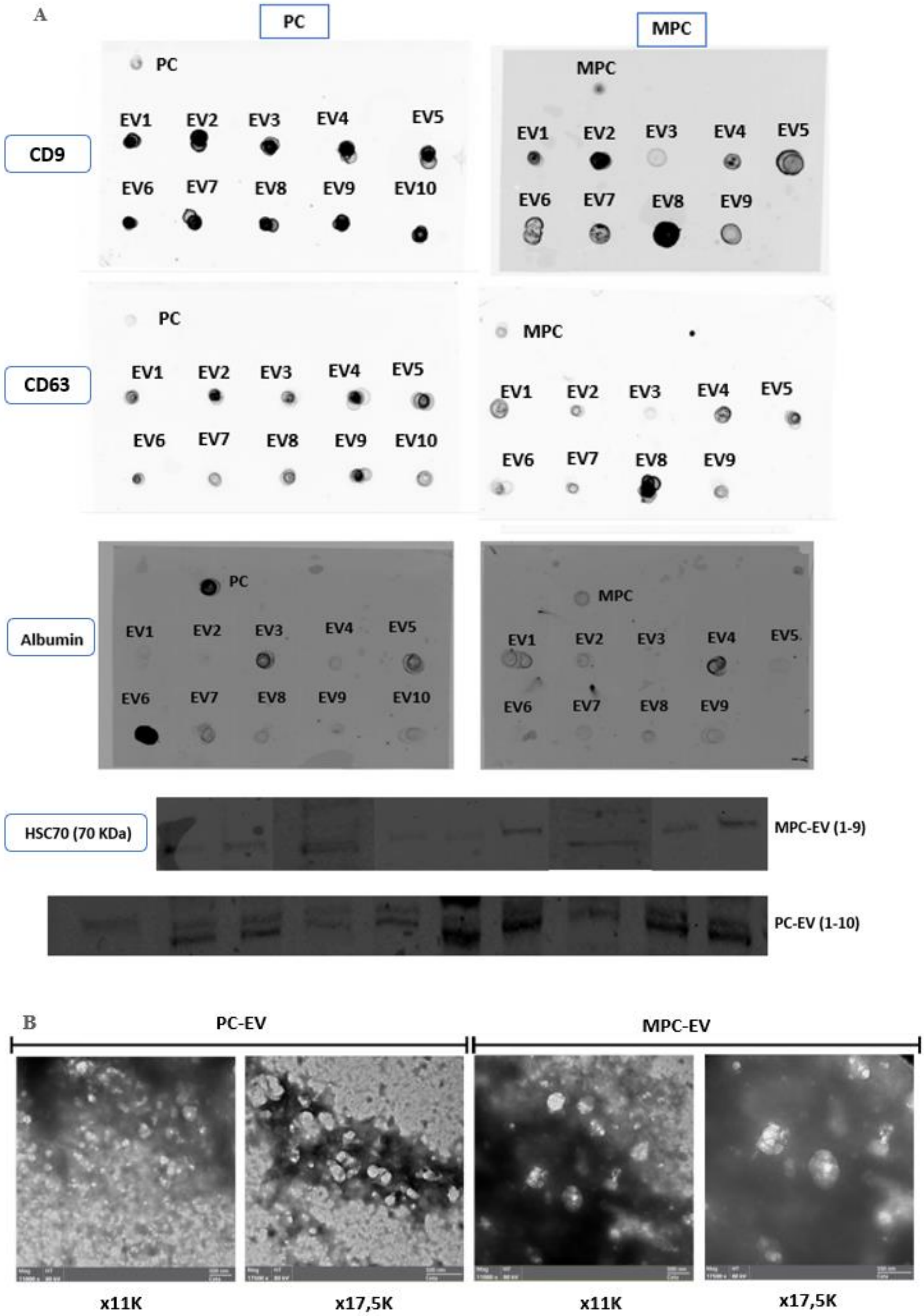

Supplementary Figure 1. Characterization of pEV markers isolated from MPC and PC respective isolated pEV. (A) Platelet concentrates and respective isolated pEVs were

evaluated for the EV membrane markers CD9 and CD63, the luminal EV marker HSC70 (71KDa) and as non-EV marker we selected Albumin as expected contaminant. For CD9 and CD63 detection, the same amount of protein (2.5 µg) was loaded, for albumin detection 5 µg were loaded, and 15 µg of protein were used for the HSC70. (B) TEM images from PC-EV and MPC-EV.

A)

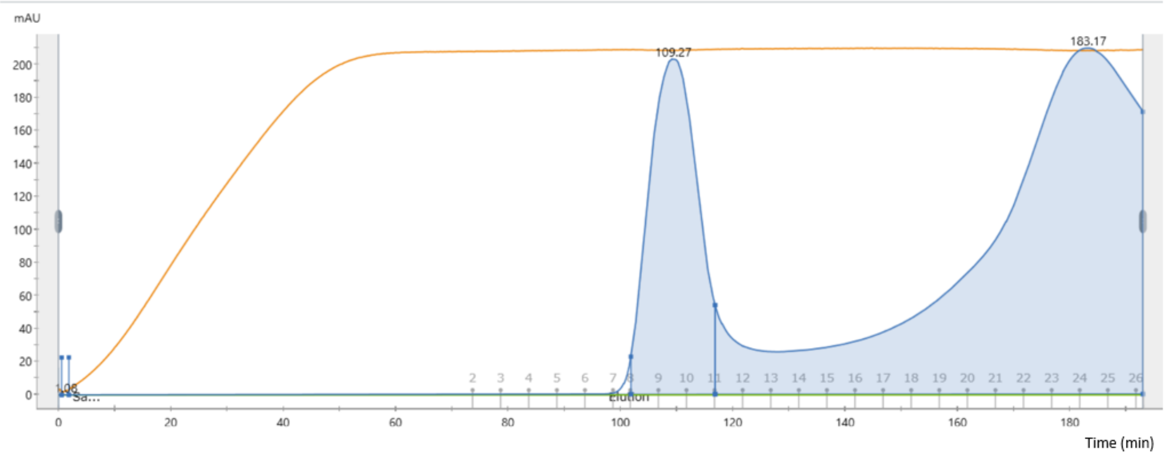

B)

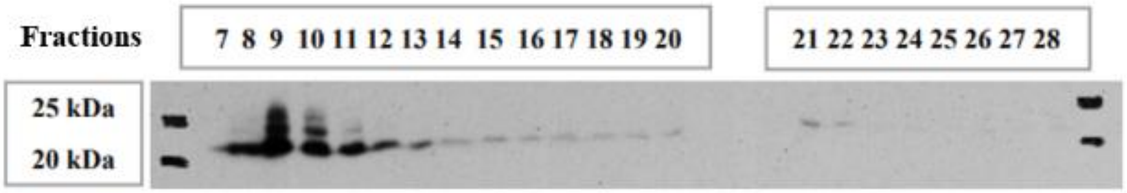

Supplementary Figure 2. SEC fraction characterization. (A) EV fraction chromatogram at an absorbance of 280 nm (blue line and area), the orange line the conductivity of the solute, and on the X axis the elution time. The numbers from 1 to 26 are each of the 5 mL fractions collected for characterization. (B) WB of SEC fractions; the enrichment of fractions 8,9,10 is observed, which are the fractions used for the pEV pools from both MPC and PC.
